# Addressing Fairness, Bias, and Appropriate Use of Artificial Intelligence and Machine Learning in Global Health

**DOI:** 10.3389/frai.2020.561802

**Published:** 2021-04-15

**Authors:** Richard Ribón Fletcher, Audace Nakeshimana, Olusubomi Olubeko

**Affiliations:** ^1^ Massachusetts Institute of Technology, Cambridge, MA, United States; ^2^ University of Massachusetts Medical School, Worcester, MA, United States

**Keywords:** artificial intelligence, machine learning, bias, fairness, appropriate use, global health, ethics, medicine

## Abstract

In Low- and Middle- Income Countries (LMICs), machine learning (ML) and artificial intelligence (AI) offer attractive solutions to address the shortage of health care resources and improve the capacity of the local health care infrastructure. However, AI and ML should also be used cautiously, due to potential issues of fairness and algorithmic bias that may arise if not applied properly. Furthermore, populations in LMICs can be particularly vulnerable to bias and fairness in AI algorithms, due to a lack of technical capacity, existing social bias against minority groups, and a lack of legal protections. In order to address the need for better guidance within the context of global health, we describe three basic criteria (Appropriateness, Fairness, and Bias) that can be used to help evaluate the use of machine learning and AI systems: 1) APPROPRIATENESS is the process of deciding how the algorithm should be used in the local context, and properly matching the machine learning model to the target population; 2) BIAS is a systematic tendency in a model to favor one demographic group vs another, which can be mitigated but can lead to unfairness; and 3) FAIRNESS involves examining the impact on various demographic groups and choosing one of several mathematical definitions of group fairness that will adequately satisfy the desired set of legal, cultural, and ethical requirements. Finally, we illustrate how these principles can be applied using a case study of machine learning applied to the diagnosis and screening of pulmonary disease in Pune, India. We hope that these methods and principles can help guide researchers and organizations working in global health who are considering the use of machine learning and artificial intelligence.

## Introduction

### Machine Learning vs. Artificial Intelligence

The advent of computing machines has enabled automation and accelerated productivity. However, human tasks that involve higher-level thinking, such as abstraction, understanding, or creativity, remain a challenge for machines. Outside of the technical literature, the terms *artificial intelligence* and *machine learning* are loosely defined and often used interchangeably; however, it is important to note that machine learning is generally considered to be a sub-field of artificial intelligence (Winston and Brown, 1984).

*Machine learning* can be generally defined as the methods and algorithms that enable computers to make optimal decisions given a set of data; these tasks can range from simple binary classification decisions to more advanced real-time control tasks, such as driving a car or playing a video game. Machine learning methods include an increasing variety of mathematical tools and mathematical models, ranging from simple logistic regression, to neural nets and deep learning, to probabilistic methods such as Bayesian networks. While computers can be used to self-discover patterns or clusters in a set of data (*unsupervised* learning), these decision-making algorithms are commonly constructed in a *supervised* manner, using a set of labeled training data to create models that can be optionally updated and modified incrementally over time with new data, as a form of continuous learning (Liu, 2017).

Human intelligence and artificial intelligence, on the other hand, involve much more than decision-making or controls. The practice of medicine also extends beyond solving diagnostic puzzles or performing robotic surgery, and also includes complex tasks such as intuitively communicating with a patient, understanding a patient’s story, expressing empathy through speech, touch, or gaze, and inferring higher-level abstractions, associations, and meaning from a patient encounter. Such tasks extend beyond the current ability of computers and remain an active area of research within the academic fields of computer science and artificial intelligence. In the context of medicine, machine learning is currently limited to tasks such as decision support (disease diagnosis and screening), processing patient medical data (e.g., detecting abnormalities in an X-ray or fundus image), or optimizing processes and services in the delivery of health care, in order to increase system capacity, allocate resources, or minimize financial losses (Beam and Kohane, 2018; Ngiam and Khor, 2019).

### The Benefits and Risks of Applying Artificial Intelligence in Global Health

Global health represents a great opportunity for employing artificial intelligence or machine learning as part of digital health initiatives (Labrique et al., 2020). Smaller clinics in LMICs often find themselves understaffed and overburdened, compounded by a lack of education. Despite a shortage of resources in these areas, the increasing presence of computers – especially smart phones – has now provided a viable platform for hosting and deploying machine learning tools. Global health researchers and the health ministries in many LMIC countries have begun exploring various ways that computers can be used. Such tasks include assisting young unexperienced doctors and health workers to perform better disease diagnosis and helping to analyze medical data, such as automatically identifying malaria parasites in a digital microscope or automatically identifying signs of coronavirus in a chest X-ray image (Elgendi, 2020). Machine learning can also be used to help optimize processes or to predict human behavior (Hosny and Aerts, 2019; Schwalbe and Wahl, 2020; Shah et al., 2020).

Without a regulatory and legal safety net, however, the deployment of any new technology in poor or uneducated regions must be carefully examined. With increasing use of computers and smart phones in health care, it is important to thoroughly examine potential harms. While machine learning is now being applied to medicine throughout the world, this issue is particularly sensitive in LMIC countries, where we often encounter populations that are very poor or politically marginalized and are very vulnerable to exploitation or discrimination. In many developing parts of the world, the legal and regulatory framework may not be well-developed for the practice of medicine and public health. As a result, artificial intelligence might only serve to reinforce and exacerbate problems stemming from socioeconomic disparities or possible political corruption. Unless the adoption of this technology is done carefully and thoughtfully, the use of artificial intelligence may simply exacerbate existing health disparities among different demographic groups.

### Basic Classification and the Connection to Ethics

**FIGURE 1:**
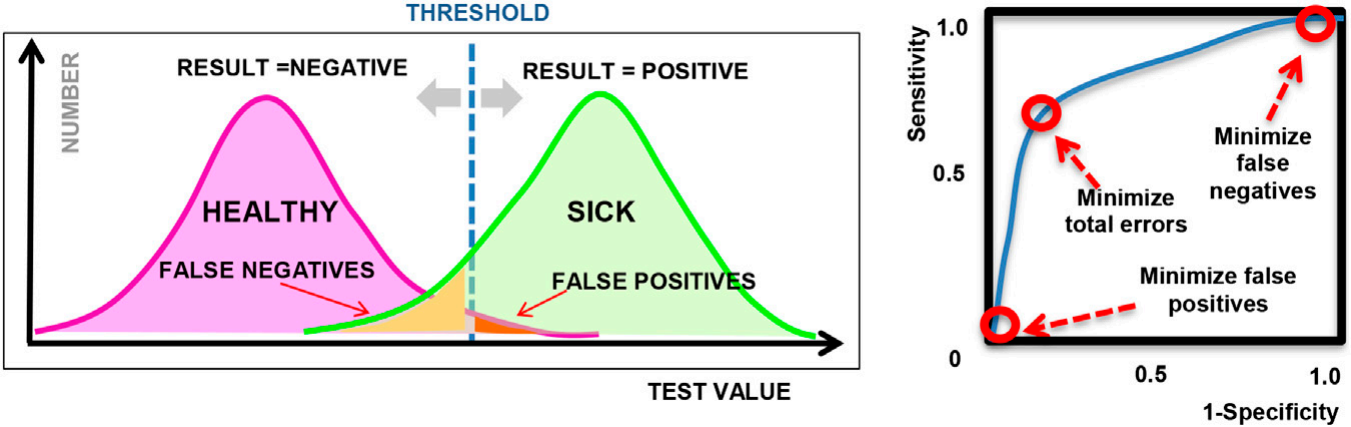
**(left)** Illustration showing sample diagnostic test results for a given threshold; **(right)** Sample ROC curve showing how the sensitivity varies for different threshold values.

A common application of machine learning in medicine is to perform binary classification based on clinical observations or laboratory measurements, which are often used in diagnostic testing or disease screening to classify individuals as *healthy* vs. *sick*, for example. While the connection to ethics may not be immediately apparent, it is important to remember that diagnostic decisions are not always correct. As we know, a medical decision, such as a diagnostic test, can produce a false positive (Type I error) or a false negative (Type II error), as illustrated in Figure 1; however, we can control the threshold used in these decisions and the relative balance between Type I and Type II errors.

The connection to ethical and legal considerations is often introduced when we discuss how to address the classification errors and how to tune or choose the *operating point* of the algorithm. Depending on the type of machine learning model used, the output of a model can generally be tuned by adjusting a threshold or certain hyper-parameters in the model. By varying the threshold or hyper-parameters, we can adjust the proportion of Type 1 and Type 2 errors, as visualized in a *Receiver Operating Characteristic* plot or “ROC curve” as shown in Figure 1 (right), where the *sensitivity* and *specificity* represent the true positive rate and true negative rate respectively, for different settings of the hyper-parameter. Many machine learning examples optimize for classification accuracy and select the point on the ROC curve that is closest to the upper left corner (as an attempt to maximize both sensitivity and specificity), or maximize the overall area under the ROC curve (AUC). However, in practice, the choice of operating point depends on the context in which the model will be used and often depends on the system of laws and ethical principles that exist in the local community.

As a simple example, we may compare a diagnostic test for strep throat (streptococcal pharyngitis) vs. a diagnostic test for the AIDS virus. In the case of strep throat, we may prefer to tune our model to minimize *false negatives*, in order to make sure that we detect all cases of the disease. The public is not alarmed by false positives because this is a very common and mild disease, and we routinely use antibiotics as a prophylaxis. On the other hand, in the case of an AIDS test, a positive result could produce great social stigma and psychological burden for the patient; so for this reason, as well as to minimize legal liability, health clinics may choose to minimize *false positives*, at the expense of missing some actual cases of AIDS. This trade-off between false positives and false negatives is a common source of debate in medicine and is frequently discussed in the context of breast cancer screening (Gøtzsche, 2012), for example. A set of legal and ethical principles must be identified prior to deciding on the preferred balance of false positives and false negatives.

### Machine Learning in the Context of Demographic Groups

The ethical component of Machine Learning becomes increasingly complex when demographic groups are introduced into the analysis. Since disease etiologies can have a known biological dependence on race or gender, it may not be surprising that the accuracy of a diagnostic test can also depend on race or gender. In situations when a given diagnostic test performs better on one demographic group vs. another, the diagnostic test may often be deemed *unfair*.

**FIGURE 2:**
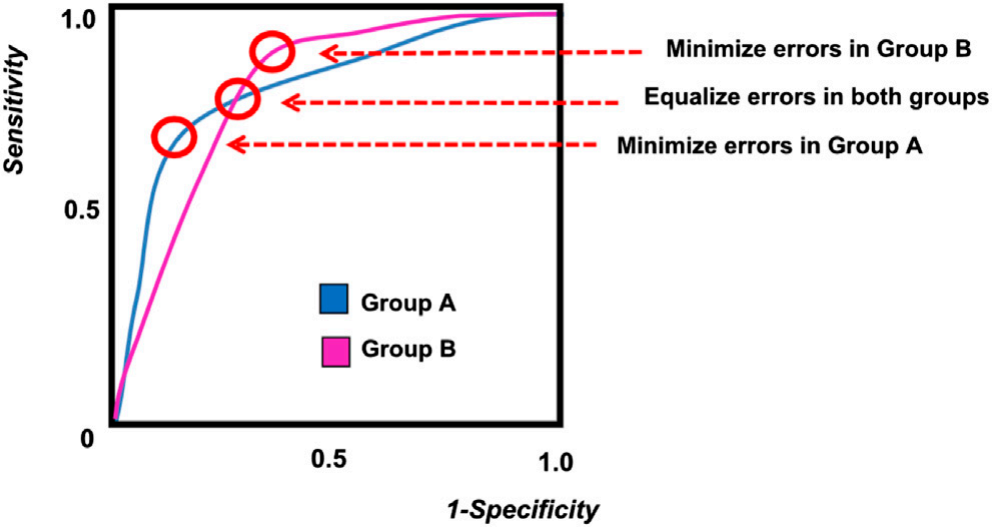
A sample ROC curve for multiple groups, showing different possible operating points for the algorithm.

When two separate demographic groups are being included in a machine learning diagnostic model, the difference between the two groups can be examined on an ROC curve, as shown in Figure 2. Due to biological differences, as well as difference in disease prevalence, each demographic group will have a separate ROC curve. As shown in the figure, it is impossible to tune the model and select an operating point that will perform optimally in both demographic groups. If a single diagnostic test will be applied to the entire population, then some compromise must be adopted in the tuning of the algorithm in order to address issues of fairness. These trade-offs are discussed more quantitatively in later sections of this paper.

In the global health and public health context, machine learning is often used with a wide variety of data including images, questionnaires and clinical measurements. In addition to biological and genetic causes, other factors may also unintentionally contribute to consistent differences between test results from two demographic groups. Examples may include cultural and behavioral differences (e.g., diet, smoking behavior, etc.), environmental factors (e.g., sanitation, exposure to air pollution, etc.), or confounding variables in the design of the measurement apparatus itself. If an algorithm is to be applied to a general population, it is necessary to consider the tuning of the algorithm to ensure fairness to all demographic groups. These decisions again depend on the prevailing legal and ethical framework in a given community.

In the context of medicine, another potential risk to patients is that new algorithms, like new medicines, are not always tested or trained on all demographic groups. In some LMIC and developing countries, certain demographic groups may hold greater political power and thus the data that is used to train or test an algorithm may come from the demographic group that is most convenient to use, or that represents the majority. This situation can produce diagnostic tests that perform better on one demographic group at the expense of others, and is thus deemed unfair.

While the eventual optimal tuning of an algorithm can thus depend on many factors, including the local system of laws and community values, the overall goal of this paper is to introduce and define the notions of bias, fairness, and appropriate use, as it pertains to machine learning in global health, and also to illustrate how a given machine learning model can be analyzed to identify and quantify issues of bias and fairness. With this goal in mind, this paper is intended for the audience of people who work in global health and international development, for the purpose of helping to develop best practices in the adoption of machine learning and artificial intelligence.

## Fundamental Considerations in the Application of Machine Learning

In the context of health applications, we discuss below three fundamental criteria (Appropriateness, Bias, and Fairness) that can be used to evaluate or audit artificial intelligence and machine learning algorithms.

### Appropriateness

While most technical literature is devoted to improving the performance of machine learning algorithms, there is little guidance available regarding how an algorithm should be assessed and evaluated prior to deployment in a real-world operational context. To meet this need, we include here an additional criteria termed *appropriateness*, which addresses how well the machine learning algorithm is matched to the specific context and the specific population.

#### Matching the Algorithm to the Specific Problem

When a machine learning model is being used as part of a public service, such as the delivery of health care, it is important to consider how and why the technology is being used. Given the increasing availability of machine learning software, it has become quite popular to try applying machine learning to many different decision-making needs in health care. However, given the limitations of artificial intelligence, it is also important to consider at the outset why a computer algorithm is being used and what is the intended role of the computer algorithm. If the consequences of a decision are very great (e.g., deciding whether or not to remove a patient from a life support machine), then it should be considered whether this decision should be handled by a machine or if perhaps a human should be part of the decision process.

In the deployment of a machine learning algorithm, it is important to consider the context. For example, it is now technically possible to install an eye scanning machine as part of an airport biometric security check that could detect certain types of cancer. However, this is not the appropriate setting for such an algorithm, even though the hardware may be available. Similarly, as we create more powerful instruments and machine learning tools for community health workers, we also need to consider what tests are appropriate to be administered by a community health worker, given the level of training and given the availability of therapeutic options. Such questions also require serious moral and ethical consideration.

A well-known example of machine learning applied in an actual hospital environment was published by Caruana in 2015 (Caruana et al., 2015). This example involved a study in the 1990s that created a computer algorithm to decide which pneumonia patients arriving at the hospital should be admitted and which patients should be permitted to go home for outpatient care. The original algorithm predicted the 30-day probability of death for each patient, and those with a higher probability would get admitted to the hospital. At the outset, this seemed like a very reasonable application of machine learning.

Unfortunately, the results of this algorithm were problematic. The computer algorithm determined that patients with comorbid respiratory ailments, such as asthma or COPD or chest pain, had a lower probability of dying, and therefore, these patients should not be admitted to the hospital and should be sent home. Based on historical data, the algorithm also recommended that patients without co-morbidities had a higher risk of death and should be admitted to the hospital. The result was the opposite of what an experienced human doctor would recommend: patients with co-morbidities and disease history should be admitted, and patients with simpler cases should be allowed to go home.

Upon re-analysis of this data, it was revealed that arriving pneumonia patients with co-morbid respiratory ailments did indeed have a statistically lower probability of death, but this was due to the fact that these patients had more experience with respiratory problems and thus sought medical care sooner, which decreased their probability of dying. But this reasoning ignored the fact that such patients are also more vulnerable and fragile in terms of developing complications and can experience more severe illness. The difference in the health-seeking behavior between these two groups was not considered, and this resulted in an incorrect prediction that could affect outcomes.

From this example, we can see that the problem was not the algorithm per se, but rather the *context* of the algorithm and the specific question that the algorithm was asking. While the probability of death may be a very reasonable question to consider for a health insurance company, it was perhaps the wrong question to ask in this context. A better question to ask would be perhaps to ascertain the severity of the infection, and based on the severity and risk factors such as age and co-morbidities, the algorithm could then recommend which patient should be admitted to the hospital. The available patient data, such as the level of fever (temperature) and comorbid respiratory ailments could have also been used to predict the severity of infection and risk of complications, but this was not done.

This example reveals not just the need for proper algorithm design and choice of data, but also identifies the need to include people with domain knowledge in the algorithm design process. At the time the algorithm was being developed, some consultation with a pulmonologist may have avoided the issues with the original algorithm.

#### Unbalanced Data and Adapting the Model to the Specific Population

When applying machine learning to problem in global health, it is important to consider the type of model as well as how the model is trained. For use in diagnostic support, the *interpretability* of the model is often highly desired by doctors, and models such as logistic regression and Bayesian Networks are quite popular for that purpose. Neural net methods, such as Deep Learning, are less interpretable but very powerful and particularly useful for data that contain abstract or hidden features, such as analysis of patient breathing sounds (Chamberlain et al., 2016b) or tumor detection in radiology images (Al-Antari et al., 2018).

With regards to training the model, a common machine learning task in global health is classification, in order to identify people that may have a specific disease (e.g., cervical cancer) or to identify people that practice certain behaviors (e.g., breastfeeding). In many cases, however, due to practical reasons, the data used to train such algorithms is often *unbalanced* (unequal numbers in each class); in this case, the smaller, minority class is often poorly predicted, although the classification acccuracy and specificity could be quite good. For example, in a diagnostic test where 99% of the patients are negative and 1% are positive, a diagnostic algorithm could trivially achieve 99% accuracy by simply predicting all patients as negative, although the sensitivity in this case would be zero. As mentioned previously, the proper balance between sensitivity (true positive rate) and specificity (true negative rate) is ultimately a subjective decision that depends on the application objectives and relies on the ethical principles being adopted.

Furthermore, when a model is going to be deployed in a particular population, the model needs to be tuned to reflect the prevalence of each class label in the specific population. For example, if a model is trained using data from one geographic region (north India), then the model may need to be modified in order to apply it in another geographic region (e.g. south India). Failure to tune the model to a specific population can result in increased misclassification errors, which would confound the ability to perform any analysis of bias or fairness.

There are several well-known approaches that are used for correcting models given an unbalanced data set, which generally depends on the type of model used. However, the most common methods are summarized below:

- *Resampling:* If a data set is large, it may be possible to randomly undersample the majority class without losing significant information in order to produce equal class sizes. However, when there is not an excess of data, a more common approach is to oversample the minority class. Popular algorithms, such as SMOTE (Chawla et al., 2002) can be used to intelligently randomly synthesize new data to grow the minority class without producing overfitting. However, in the case of health applications, the data is commonly comprised of questionnaire data and medical record fields which may be binary [e.g. “Does the patient have a fever? (YES/NO)”] and are difficult to synthesize without producing overfitting.
- *Ensemble methods:* Two or more types of models can be combined to produce a hybrid classifier that performs well on the unbalanced data. Approaches include *bagging* techniques (bootstrap aggregation), to reduce variance and overfitting, and *boosting* methods, to reduce bias. Popular ensemble methods include Adaboost and Extreme Gradient Boost (Galar et al., 2011). While ensemble methods may work well for challenging tasks such as cancer detection in radiology, it is less preferred in diagnostic tasks due to a lack of interpretability and increased complexity.
- *Cost function and hyper-parameter tuning*: a simpler method that is popular for common types of machine learning models is to tune the hyper-parameters or cost function that is used for optimization in the training of the model. The penalty for misclassifying a member of the minority class can be increased by adjusting the class weights, which is a standard practice in logistic regression.
- Combinations and variants of these methods also exist, such as SMOTE-Boost (Chawla et al., 2003), which combines Adaboost with intelligent oversampling.

When tuning the hyperparameters of a model, the proper optimization criteria should be chosen in order to ensure that the minority class is properly predicted. Instead of maximizing classification accuracy,

$$Classification\;Accuracy=\frac{TP+TN}{TP+TN+FP+FN}$$

other criteria such as the F_1_ score can be chosen,

$$F_{1}=\frac{2TP}{2TP+FP+FN}$$

which penalizes more heavily the cost of false negatives and false positives (Raschka, 2014).

More recently, other measures, such as the Matthews correlation coefficient (MCC) (Chicco and Jurman, 2020), are also being used as a preferred optimization criteria for evaluating binary classifiers when a class imbalance exists.

In addition to class imbalance, a model also generally needs to be tuned to the specific population where it is being deployed. For example, the prevalence of tuberculosis among hospital walk-in patients is generally different than the prevalence of tuberculosis in the surrounding community.

In probabilistic methods such as Bayesian Networks, the true prevalence of each class is accounted by adjusting the Bayesian priors. In logistic regression, however, the operating point can be tuned by simply adjusting the zero-order term in the predictor function as follows:

Given a logistic regression prediction model, Y,

$$\hat{Y}=\beta \cdot X_{i}=\;\beta_{0}+\beta_{1}x_{1}+\;\beta_{2}x_{2}+\;\beta_{3}x_{1}+etc.$$

We can modify β_0_ as follows to create a new modified model Y^*^:

$${\hat{Y}}^{*}=\;\beta_{0}^{*}+\beta_{1}x_{1}+\;\beta_{2}x_{2}+\;\beta_{3}x_{1}+etc.$$

Where

$$\beta_{0}^{*}=\;\beta_{0}+\;\log\frac{\pi }{1-\pi }-\log\frac{\hat{\pi }}{1-\hat{\pi }}$$

π= actual prevalence in the population and $\hat{\pi }=\;prevalence\;in\;the\;training\;data$

### Bias

#### Defining Bias

Since computers are psychologically associated with pure impartial logic, it was often thought that computers could be an ideal solution to the human problem of discrimination and prejudice in complex decisions. We now know, however, that machine learning algorithms and the data used to train them are generally a product of human design and can also be flawed.

The term *bias* has multiple definitions, depending on the context; however in terms of algorithms, we can define bias as a systematic error or an unexpected tendency to favor one outcome over another (Mehrabi et al., 2019). The term bias is also used to describe when an algorithm has an undesired dependence on a specific attribute in the data that can be attributed to a demographic group. An ideal unbiased algorithm should not be dependent on any protected attributes of a patient, such as gender, race, or religion. If algorithmic bias leads to unfavorable treatment of one patient group vs. another, this bias can be judged to be unfair, from a legal or ethical point of view.

While bias is related to *fairness*, it should be noted that algorithmic bias is independent of ethics, and is simply a mathematical and statistical consequence of an algorithm and its data. If an algorithm is discovered to have bias, this bias can then be judged against a set of ethical or legal principles to determine if unfairness exists, and then the tuning of the machine learning model can be adjusted to satisfy certain fairness constraints. The issue of fairness is discussed in the next section of this paper.

While some researchers have identified more than 20 different types of bias (Mehrabi et al., 2019), the various types of bias can be more simply categorized as having *implicit* or *explicit* causes. These causes may include problems with data sampling, ideosyncracies of the algorithm, the design of the equipment used to collect the data, or unexpected human behavior in the data collection process. The most common form of bias is sampling bias, resulting from the use of unbalanced or missing training data that do not adequately reflect the actual proportions found in the real world. A more challenging form of bias, however, is implicit bias that is due to unforeseen correlations between variables in a model. As a result, it is standard practice to avoid patient data fields that may correlate with protected attributes, such as a race, gender, or religion. For example, machine learning algorithms should not explicitly discriminate against low-income patients, but if the algorithm uses a patient’s home address or postal code, this variable may also correlate with the patient’s socioeconomic status and generate implicit bias with respect to the patient’s socioeconomic status.

#### Examples of Bias in Machine Learning

The existence of systematic bias is well-known in the bioinformatics field, and often occurs in the high-throughput processing of microbiological specimens for testing (the so-called “batch effect”), due to small systematic differences in the machines or environmental conditions (Papiez et al., 2019). The connection of bias to fairness attracted publicity in recent years due to the discovery of systematic biases that can impact specific demographic groups. A well-known example of machine learning bias, publicized by Joy Boulamwini in 2017 (Buolamwini, 2017), was the performance of facial detection algorithms when applied to people of different skin colors. It was shown that facial detection models created by IBM and Microsoft at the time performed surprisingly poorly (accuracy <40%) when applied to dark-skinned women. Since race and facial features are protected attributes, this problem raised the issue of fairness; however the main cause was the image features employed in the face detection algorithm. This algorithmic bias could have been greatly reduced, for example, by increasing the dynamic range of the image to reduce the contrast and including a larger proportion of dark-skinned faces in the model training.

In another example from 2016, it was reported that computer algorithms that process passport applications were rejecting some Asian applicants because their passport photo was determined to have their eyes closed (Regan, 2016). The algorithm trained on Caucasian eyes was not able to properly process facial images from Asian people and incorrectly rejected their photos.

While physical features may be an obvious example of differences that can produce bias, a more subtle issue is the problem of unforeseen biases that result from cultural or socio-economic factors that are endemic to a specific population. For example, some researchers have used data from the famous Framingham Heart study, which mainly included white males, to develop a cardiovascular risk prediction algorithm for African-American patients; and the result was not successful because the two demographic groups have different risk factors and disease etiology (Gijsberts et al., 2015). It cannot be generally assumed that an algorithm trained with data from one demographic group can be used to develop a prediction algorithm for another demographic group. In practice, these problems may be difficult to uncover when an algorithm is improperly advertised and the training data for the algorithm are not disclosed to the public, customers or the patients.

#### Mitigation of Bias

If bias is discovered in an algorithm, a variety of solutions are now available to help mitigate and reduce the level of bias (Bellamy et al., 2019). For algorithms that involve image processing or signal processing, bias can often be mitigated by examining the processing algorithm and changing the way the data is analyzed and the way the features are extracted. Bias can also be addressed at the data collection stage by adjusting the sampling process (Kamiran and Calders, 2012; d’Alessandro et al., 2017) and making sure that all classes are properly represented in the training data. Algorithmic bias can also be addressed in the prediction algorithm itself through the use of custom regularization constraints or cost functions which determine the relative “cost” of making an incorrect decision (Kamishima et al., 2012) or adversarial learning algorithms can be used to minimize the bias (Zhang et al., 2018). Bias can also be addressed at the output stage by adjusting how certain outputs are labeled (Hardt et al., 2016). Despite these emerging methods to mitigate bias, the presence of bias in an algorithm often goes undetected. Therefore, it is important, as standard practice, to develop methods to test for bias at all stages of algorithm development. A simple example of this process is presented as a case study in this paper.

It is also important to note that bias is not necessarily problematic. As an example, a published study from Rwanda (Fletcher et al., 2021) demonstrated how a computer algorithm could help identify an infection in a surgical wound, just using a color photograph of the wound. While the machine learning algorithm was reasonably successful among dark-skinned women, the same algorithm would likely perform poorly if used with lighter skinned women in another country such as Ethiopia or Mexico. However, this bias could be tolerated if used exclusively for dark-skinned women. Therefore, even if bias exists in an algorithm, it may still be acceptable to use such an algorithm within a specific context, provided that the limitations and appropriate use of the algorithm are properly declared, disclosed, and documented.

### Fairness

A major consideration of a decision-making algorithm is the fairness of its decisions. Unlike bias, the *fairness* of a machine learning model is judged against a set of legal or ethical principles, which tends to vary depending on the local government and culture. In addition to diagnostic prediction, machine learning algorithms are now being applied to operational aspects of health care delivery, such as decisions regarding admissions and triage, as well as determining the cost of insurance premiums that a patient should pay. All these applications have the ability to produce unfair outcomes with respect to demographic groups; therefore, it is necessary to have a framework for quantitatively assessing the fairness of such decisions. The following sections provide an overview of different ways fairness can be defined and measured, to enable the proper tuning of a machine learning algorithm.

#### Individual vs. Group Fairness

Given the dictionary definition of fairness (impartial and just treatment), we can consider fairness at the level of an *individual* or a* group*. We can ask whether a computer algorithm disproportionately helps or harms specific individuals or specific groups of people.

Ideally, an algorithm would be customized to an individual, and fairness criteria could be satisfied by ensuring that the algorithm provides similar treatment to individuals that share similar characteristics. Mathematically, if it were possible to describe an individual by a set of parameters in a multi-dimensional space, then all individuals within the neighborhood of the same parametric space would be treated similarly and receive similar predictions from a machine learning algorithm (Zemel et al., 2013). This type of fairness is known as individual fairness and is a measure of *consistency*. This topic remains an active area of research.

In practice, however, many countries contain laws that regulate fairness and prevent discrimination in terms of specific demographic groups, defined by race, gender or socioeconomic status. Furthermore, from a biological perspective, we also know that the prevalence of certain diseases tends to vary across different racial and ethnic groups. (e.g., diabetes in the South Asian population, or hypertension in the African-American population). We also know that there exist significant physiological variations across racial and ethnic groups (e.g., South Asian people have smaller average lung capacity than Caucasian European people). In some cases, diagnostic tests use a different criteria for different ethnic groups (e.g., the normalized standards for spirometry used in pulmonary function testing); but other types of machine learning models are applied equally across multiple demographic groups. For these practical reasons, the implementation of fairness metrics for machine learning is generally described in terms of *group fairness*.

#### Quantifying Fairness

In general, a prediction algorithm can be in the form of a continuous regression, such as in the case of estimating a value for a given patient’s insurance premium, or the algorithm can be in the form of a classifier with a binary outcome, such as deciding whether or not to provide a specific treatment to a patient. Fairness criteria can be applied to both types of algorithms; however, for the purpose of simplicity, we choose here to describe the case of binary classifiers.

Mathematically, we can describe a binary prediction algorithm, denoted as Y, that will produce either a positive result Y = 1 or a negative result Y = 0, based on a patient’s data denoted by X. However, since the algorithm is not perfect, there will be times when the algorithm can deny treatment to a patient who deserved treatment (false negative) or can approve treatment for a patient that did not need treatment (false positive). As described previously (Figure 1), the relative ratio between these types of error can be adjusted by a threshold or tuning hyper-parameter, λ, that can be depicted graphically by the ROC curve.

When a prediction algorithm is developed for two different demographic groups, designated as G = a and G = b, it is very likely that the resulting ROC curve will be different for each group (Figure 2). The difference in the ROC curves can be due to possible differences in genetics, behavior, or environment caused by ethnic, educational, or socioeconomic factors. When it is necessary to implement a single algorithm for both groups, the question of fairness arises when determining the optimum operating point for the algorithm.

#### Definitions of Fairness

As illustrated in Figure 2, the ROC curves for the two demographic groups will generally have different false positive rates and false negative rates. While it is possible to define many different types of fairness criteria in terms of probability or statistics (Barocas et al., 2017; Kusner et al., 2017; Verma and Rubin, 2018), we describe here the three most common definitions for fairness:

- *Equal Outcomes*, otherwise known as *Demographic parity*, enforces that the outcome of an algorithm be equal for both demographic groups. In this case, an algorithm must approve treatment for the same rate of patients from Group A as from Group B. The same (positive rate) fraction of shall be approved from both groups.

Mathematically, this can be described as follows:

$$P\left(\hat{Y}=1|G=a\right)=P\left(\hat{Y}=1|G=b\right)$$

This definition of fairness satisfies a mathematical criterion known as *independence*:

Y⊥G

which enforces that the prediction algorithm Y is independent of the *protected attributes* that define each group G. This definition of fairness focuses on outcomes only, and ignores the error rates and any disparity in the error rates.

- *Equality of Opportunity* takes into account the error in classification, and enforces that the true positive rates are equal for both groups. This ensures that the positive outcomes from both groups have the same fraction of misclassification errors. Since true positive rate is also known as the sensitivity, this condition of fairness requires that both groups have the same level of sensitivity, which corresponds to a horizontal line on the ROC curve (Figure 2).

Mathematically, this can be described as follows:

$$P\left(\hat{Y}=1|Y=1,\;G=a\right)=P\left(\hat{Y}=1|Y=1,\;G=b\right)$$

Equality of opportunity satisfies a mathematical criterion known as *separation*:

$$\hat{Y}⊥G|Y$$

This definition of fairness ensures that the same fraction of patients in each group will receive correct positive test results, but it ignores any disparity in the negative error rates.

- *Equality of odds* requires that both the positive error rates and the negative error rates be equal across both groups. This is equivalent to stating that the sensitivity of both groups are equal and the specificity of both groups are also equal. This corresponds to the point where the ROC curve from Group A intersects the ROC curve from Group B (Figure 2).

Mathematically, this can be described as follows:

$$P\left\{\hat{Y}=1|Y=y,\;G=a\right\}=P\left\{\hat{Y}=1|Y=y,\;G=b\right\},\;y\in \left\{0,1\right\}$$

If calibrated, this definition of fairness satisfies a mathematical criterion known as *sufficiency:*

$$Y⊥G|\hat{Y}$$

This definition of fairness balances both the positive and negative error rates across both groups, but generally results in a lower overall classification accuracy.

The definition of fairness that is adopted depends on the local circumstances and the applicable legal and ethical principles. For example, in the case of an algorithm that decides college admissions, we may want an algorithm that produces equal numbers of admitted students from each demographic group, thus preserving demographic parity. In the case of algorithms that determine the success of loan applications, perhaps we may prefer to focus on the fraction of successful loan applicants from each demographic group, thus enforcing equality of opportunity. In the case of medical diagnostic tests, the equality of odds criterion is often chosen, since we want the test to perform equally well on individuals from each demographic group, even though the number of individuals testing positive will differ.

Furthermore, it can be shown that it is not possible mathematically to satisfy all the three fairness criteria described above, unless both groups have equal prevalence rates or the algorithm has perfect accuracy (Kleinberg et al., 2016; Miconi, 2017). While the equality of odds is perhaps the most popular choice in the medical context, if the resulting accuracy is too low, it may be preferable to construct separate models for each group. It is also important to note that in some cases, demographic parity or other definitions of fairness may be mandated by law (e.g., the notion of *equal protection*). When developing an algorithm for a specific country or location, it is thus important to investigate the local laws and include the opinions of key stakeholders in the design process.

### Preparing a Model for Deployment

The long-term adoption of any new technology in a global health setting is challenging and depends on many factors. However, the three criteria described above will help to ensure that a machine learning model will be better accepted by the local community and clinical staff. Since the training and tuning of a machine learning model can vary widely, it is possible that future use of machine learning in medicine may additionally require some disclosure on the part of the developers to state the conditions over which a model is valid, and to disclose possible applications or situations where a given model should not be used. At present, the regulatory framework for algorithms in medicine remains undeveloped.

**FIGURE 3:**
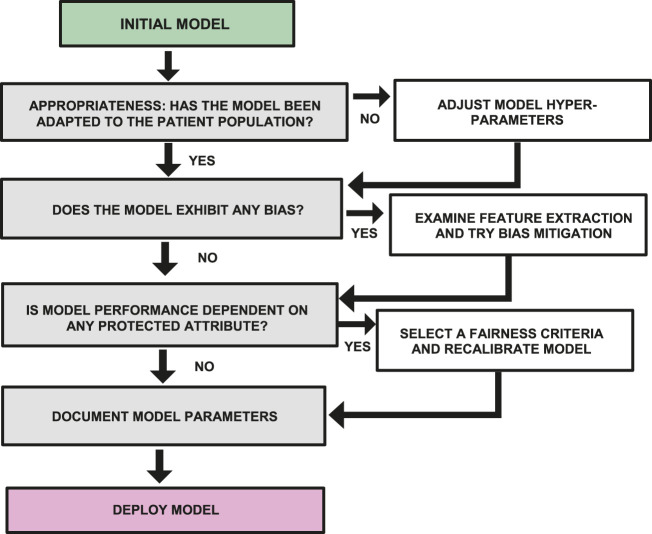
Workflow diagram showing steps required to examine a model for bias, fairness and appropriate use before deployment.

To serve as a simple guide for researchers working in global health in LMICs, it is possible to summarize the previous discussion into a workflow diagram that describes the additional analysis and tuning that should be applied to a machine learning model before it is deployed. Figure 3 represents a version of this workflow diagram.

## A Case Study: Pulmonary Disease Screening in India

In section, we present a case study in which we describe the use of machine learning applied to the problem of pulmonary disease screening, and we illustrate how the aspects of bias, fairness, and appropriate use can be addressed.

### Clinical Study Description

A study was conducted to develop and test a set of algorithms to predict several different pulmonary diseases. In LMIC countries, such as India, the burden of pulmonary disease is very high, with chronic diseases such as COPD being a second leading cause of death, and asthma representing a major cause of disability. The medical care in rural India suffers from a low doctor-patient ration of 1:1700 and includes many forms of non-allopathic medicine, which results in relatively high levels of underdiagnosis and misdiagnosis (Reddy et al., 2011). In order to address this need, machine learning algorithms were developed to help increase the capacity of GP doctors to better diagnose pulmonary diseases, using a simple set of diagnostic tools (stethoscope, peak flow meter, and smart phone questionnaire). The data used for algorithm development were collected as part of an IRB approved clinical study conducted by MIT and the Chest Research Foundation in Pune, India. The 320 study subjects ranged in age from 18 to 73 and included 87 healthy controls (Chamberlain, 2016).

The total number of study subjects for each pulmonary disease was as follows:

- 26 AR Patients
- 48 Asthma Patients
- 54 Asthma + AR Patients
- 36 COPD Patients
- 11 COPD + AR Patients
- 87 Healthy controls
- 41 Other Patients

Despite efforts made to recruit equal numbers of women and men, the data set was slightly unbalanced with respect to gender, resulting in 171 male and 132 female patients.

Machine learning models were developed to help predict the individual risk of several pulmonary diseases, including Asthma, COPD, and Allergic Rhinitis. In order to provide proper diagnostic labels and training data for the machine learning algorithm, every subject in the study was also administered a complete battery of pulmonary function tests (PFT), which included spirometry, body plethysmography, impulse oscillometry (IOS), and lung gas diffusion testing (DLCO). Based upon these tests, an informed diagnosis was given to each patient by an experienced chest physician.

### Model Development and Considerations for Appropriateness

#### Problem Definition

In order to properly develop and train our machine learning model it was important at the outset to identify the exact purpose of the model. In this case, we decided to focus first on developing a machine learning model for the purpose of decision support, to help general practitioner (GP) doctors who do not have significant experience diagnosing pulmonary diseases. With this goal in mind, it was appropriate that we use a model that is highly interpretable and that we tune our model to match the prevalence of diseases encountered at a typical local GP clinic.

#### Model Selection and Baseline Implementation

Given our need for interpretability, we elected to use logistic regression supervised learning for the study. Logistic regression enables a simple coefficient analysis to be generated that helps explain the relative weight and contribution of each feature in the model. For the purpose of this paper, the included features of the machine learning model were the questionnaire and the peak flow meter data. The questionnaire data were treated as binary variables and the peak flow meter data were normalized to the population normal and then converted to binary variables by dividing the reading into ranges (high, medium, and low).

A baseline logistic regression model was implemented using the Python Scikit-learn module, with L2 regularization. Approximately 80% of the data was used for training, and 20% of the data was reserved for testing. A separate model was created for each disease (Asthma, COPD, and Allergic Rhinitis (AR)). Since some patients had comorbid conditions (e.g., Asthma + AR, or COPD + AR), the training data for each model included only patients that had a single disease (e.g., Asthma only, COPD only, and AR only). This training methodology produced the highest accuracy as measured by AUC (area under the ROC curve).

#### Model Tuning and Correcting for Unbalanced Data

For each of the models we created, we tuned the logistic regression C-parameter by using the *grid_search* function in the Python logistic regression module, using the AUC as the optimization parameter. In order to correct for the unbalanced data, a second tuning step was then performed using the *class_weights* parameter in the Python logistic regression module to adjust the weights in the training data with higher weight given to the minority class. The F1 score was used for optimization in this step.

The resulting approximate median accuracy of the three models (COPD, Asthma, AR) had AUC values of 85, 75, and 95%, respectively.

Using these trained models, we then proceeded to analyze these models for bias as described below.

### Bias Analysis

#### General Considerations and Methodology

Using the previously trained and tuned models for each pulmonary disease, we then tested for the existence of bias with respect to gender and Socio-economic Status (SES). We examined two forms of bias, described below:

- *Systematic bias:* We first tested for systematic bias by creating two equal-size homogenous test groups (e.g., all male and all female) and then testing the AUC accuracy for each demographic group. We also observed the qualitative shape of the ROC curve for each demographic group.
- *Sampling bias:* To test for sampling bias, we kept the test set constant, but varied the training set. We created several training sets having different proportions of each demographic group, and then we checked to see if the mean accuracy and variance of the model had any dependence on the composition of the training set.

While logistic regression is a deterministic process, it is important to note that the performance of the model will generally change as the members of the training set are changed. Since the members of the training data set are randomly assigned, this produces a variance in the performance of the model every time that we conduct a new training. As a result, there is a stochastic component to the model accuracy due to these random variations in the training data, which needs to be considered (Deitterich, 1995).

In our analysis, in order to minimize the stochastic variation, and focus on the error due to bias, each bias testing configuration was run for 1,000 iterations and the mean accuracy of each model was calculated as well as mean of the resulting regression coefficients. The variation of the model output was recorded in the form of the inter-quartile range (IQR). (The variance was not used since the distribution is not Gaussian.)

#### Gender Bias Analysis

**FIGURE 4:**
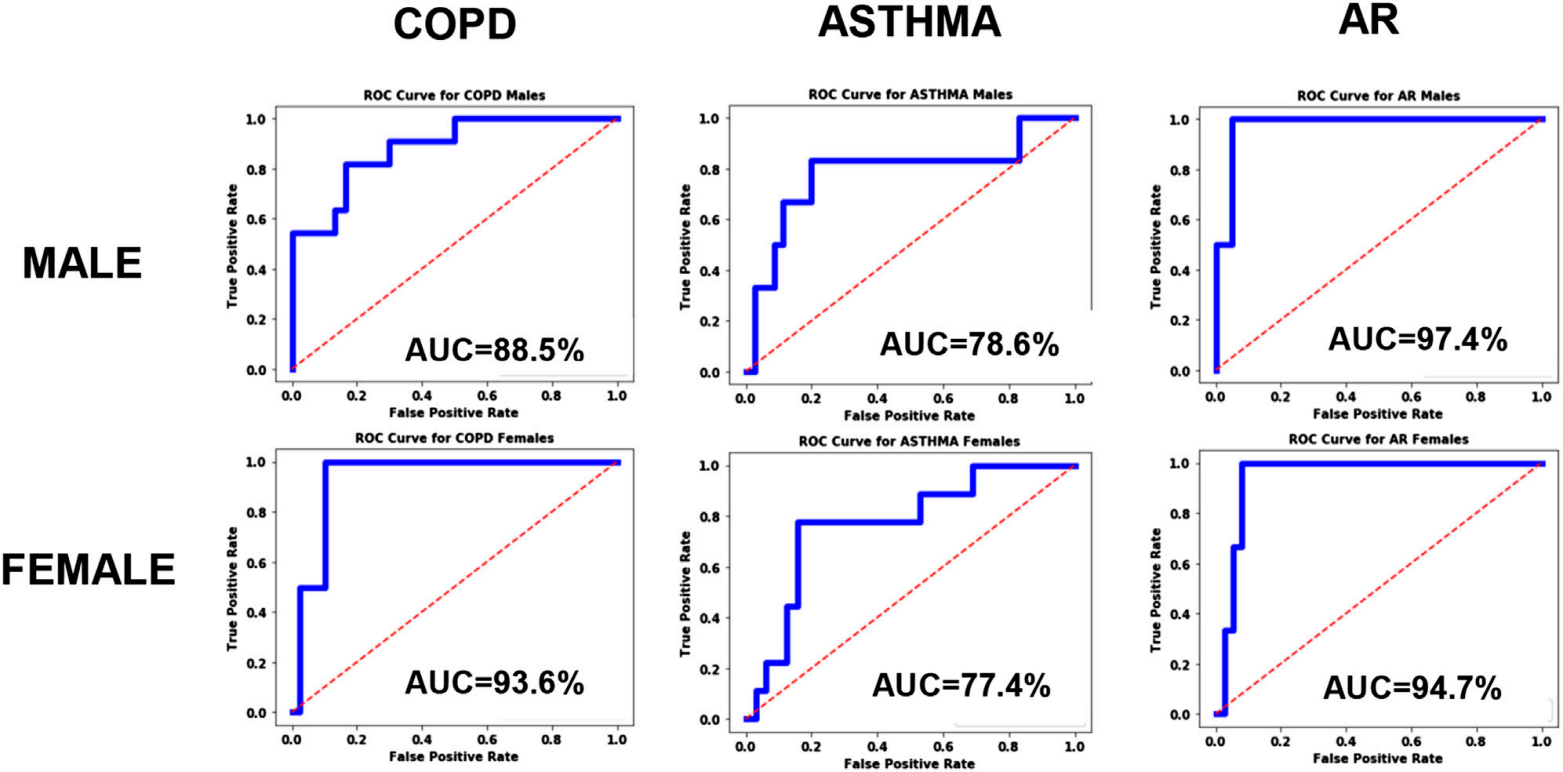
ROC curves for the disease models corresponding to COPD, Asthma and AR as a function of Gender.

As described above, the systematic bias was examined by testing the accuracy of the model using equal size homogenous training sets, and the resulting ROC curves are shown in Figure 4. From the plots we can immediately see qualitative differences between the male and female ROC curves for COPD, indicating a statistically significant gender bias, with higher accuracy for women (AUC = 93.6%) than men (AUC = 88.5%). The models for Asthma and AR, however, did not exhibit any significant gender bias.

**FIGURE 5:**
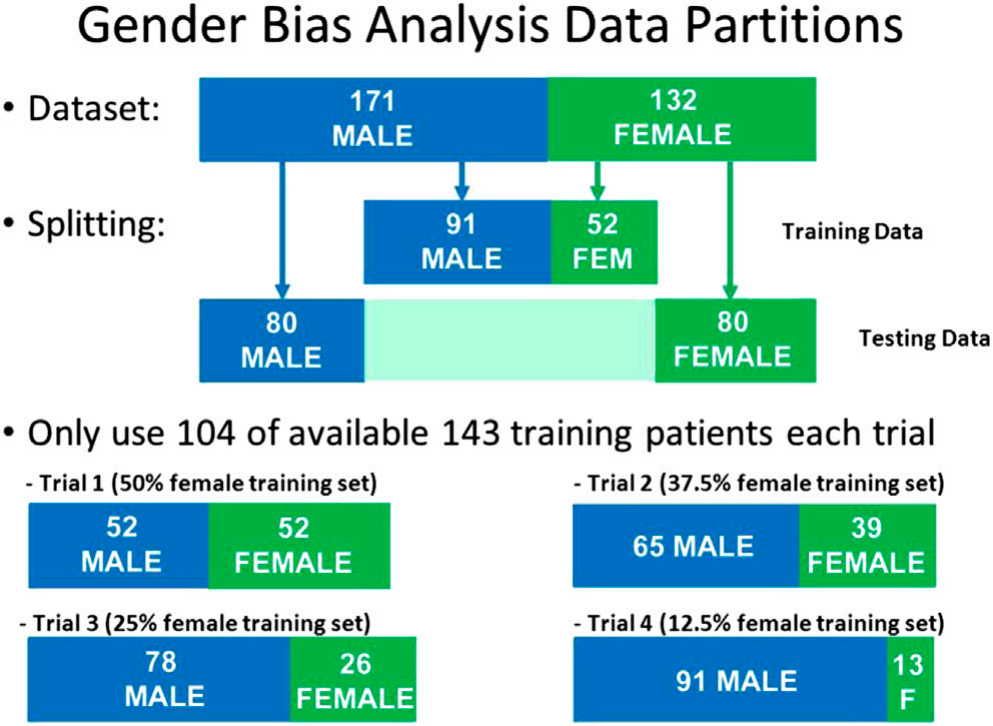
Data partitions used for Gender Bias analysis. The size of the test set and the size of the training set were kept constant, but the proportion of males and females was varied in the training set.

**FIGURE 6:**
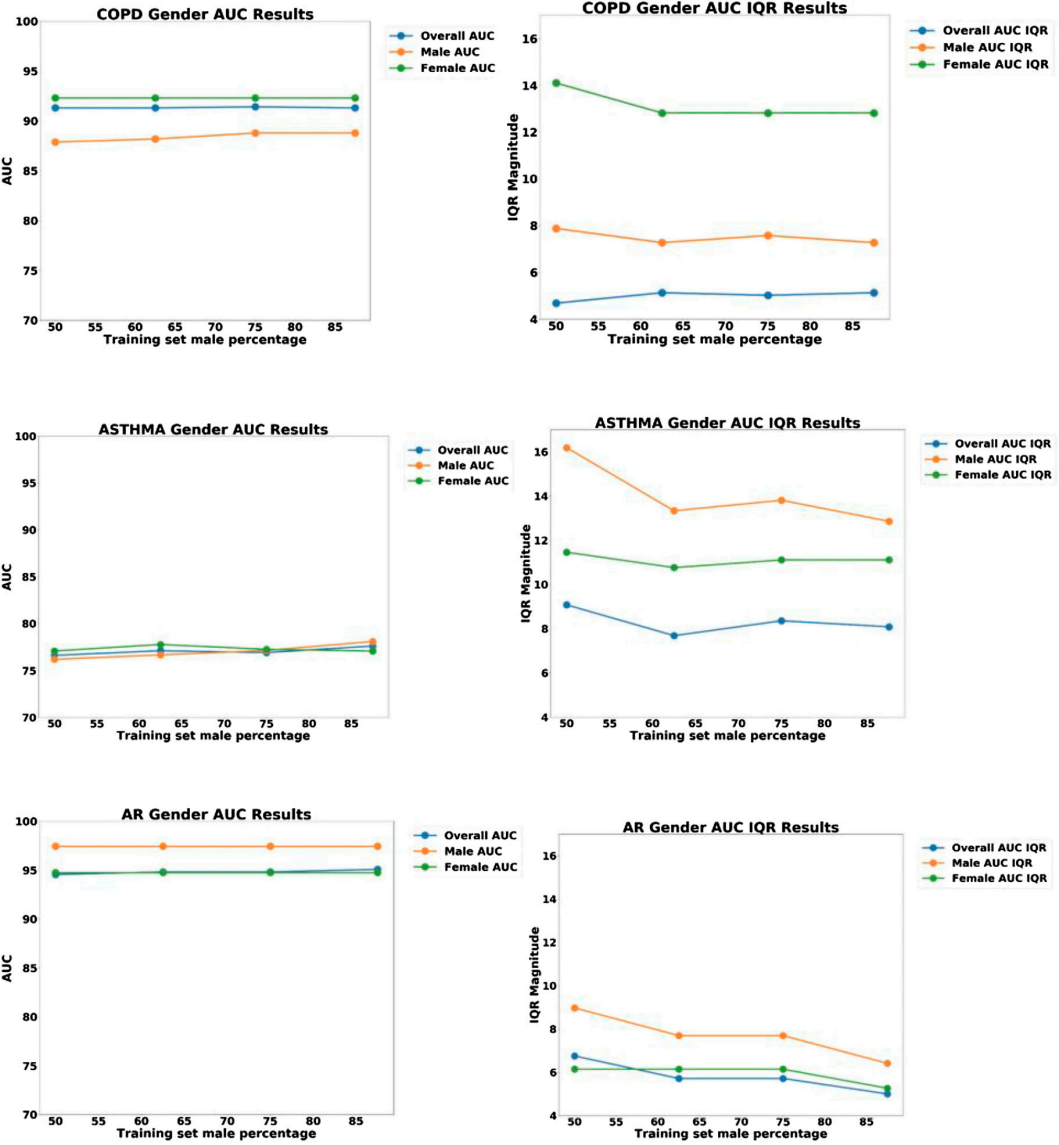
Plots of AUC Accuracy **(left)** and InterQuartile range **(right)** results of Gender bias analysis for three different disease diagnostic models: **(top)** COPD; **(middle)** Asthma; and **(bottom)** Allergic Rhinitis (AR). The three colored lines in each plot represent the AUC accuracy for all groups, male only, and female only, respectively. The horizontal axis represents the proportion of males in the training set, ranging from 50to 100%.

In order to test if this bias was due to sampling, we created four separate test sets, each of size N = 104, but having different proportions of males and females: 50% female, 37.5% female, 25% female, and 12.5% female. For each iteration of the model, a different set of patients were randomly selected to be part of this N = 104 training set. For all iterations, the same held-out test set (N = 160) was used, consisting of N = 80 males and N = 80 females. The respective data partitions for each experiment are shown graphically in Figure 5, and the results of 1,000 iterations of this analysis are shown in Figure 6 for the three disease models.

From the plots in Figure 6, we can see that the accuracy of all three models did not change significantly as a function of the proportion of women in the training set. This indicates that sampling bias is not a significant concern for these models.

However, the results do show that there is a significant systematic diagnostic gender bias between males and females for COPD, and a small systematic bias for AR. For Asthma the model performs equally well for both male and female patients.

The plots of IQR reveal that there is significant variability in the COPD and Asthma patients, with the female patients having the highest variability for COPD and the male patients having the highest variability for Asthma. For AR, there was low variability for both males and females.

#### Socio-Economic Status Bias

In order to explore potential socio-economic bias, a similar methodology was used to partition the data using pools of patients having low-SES and high-SES.

**FIGURE 7:**
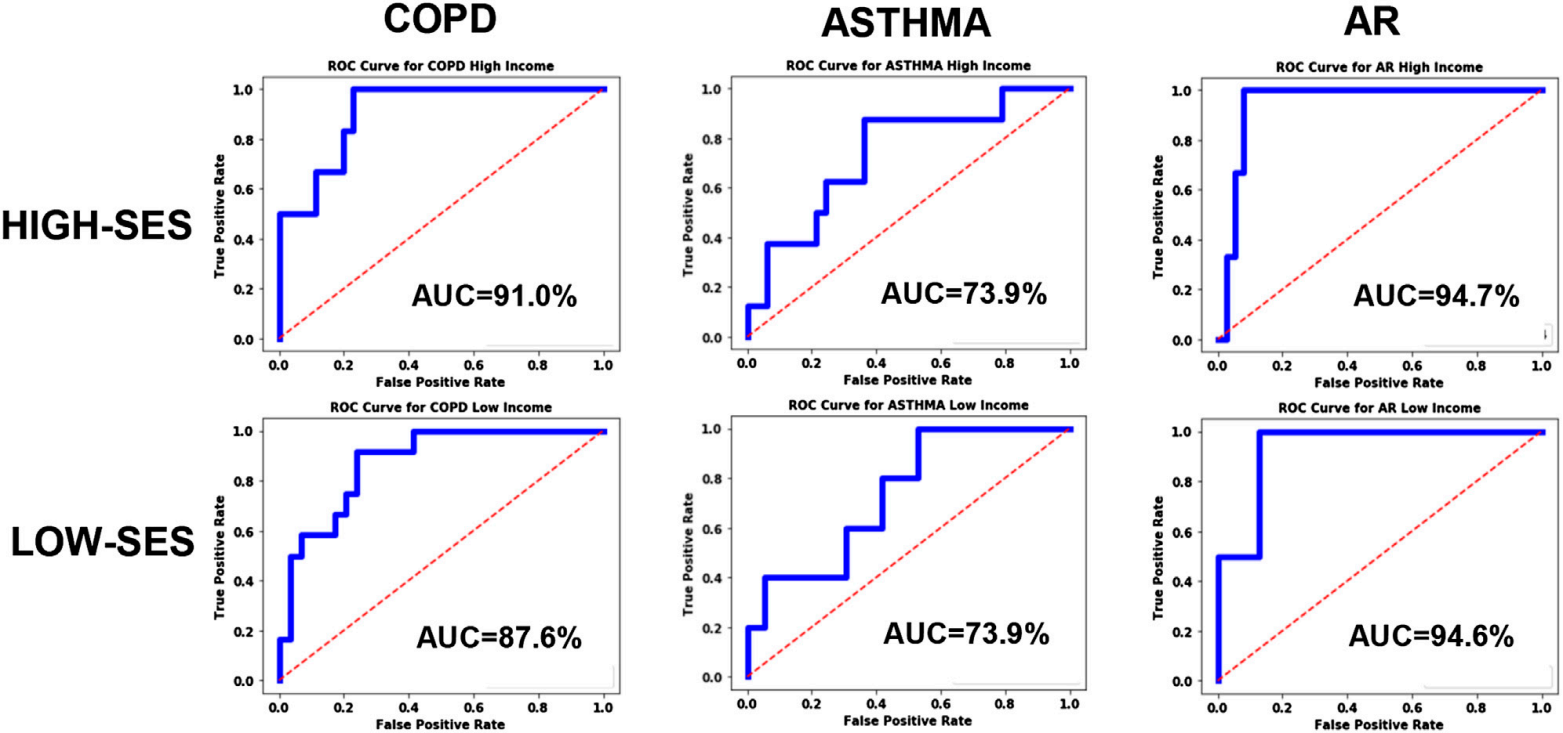
ROC curves for the disease models corresponding to COPD, Asthma and AR as a function of socio-economic status.

For exploring systematic bias, the ROC curves were plotted for homogenous pools of patients (high-SES vs. low-SES), and the results are shown in Figure 7. Asthma and AR did not exhibit any significant bias; however, COPD exhibited some systematic bias, with the high-SES group having higher accuracy.

**FIGURE 8:**
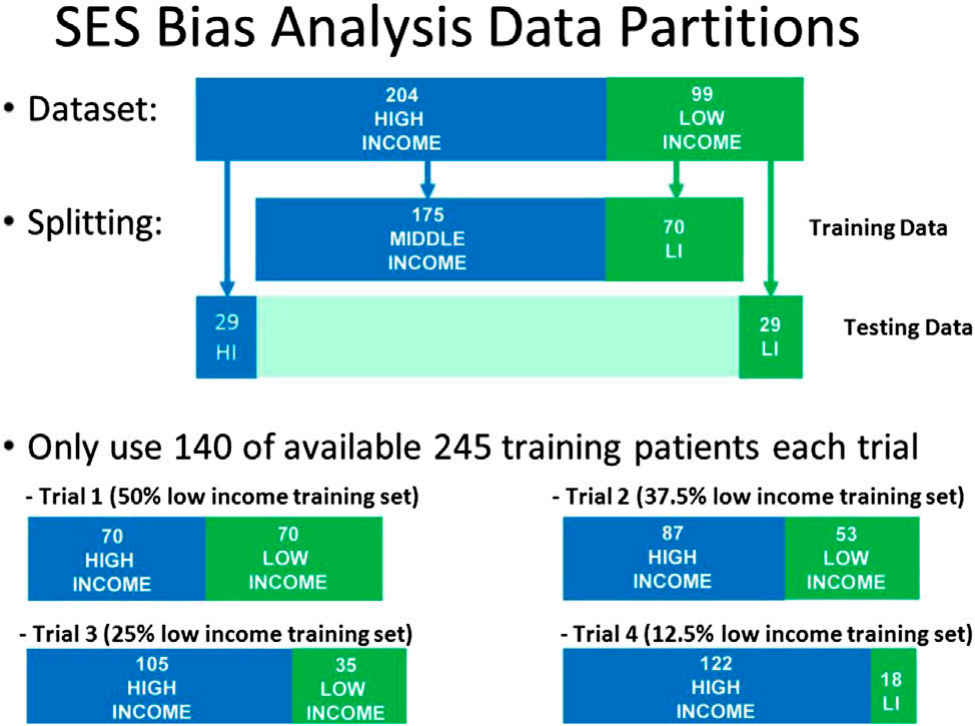
Data partitions used for Socio-Economic (SES) bias analysis. The size of the test set and the size of the training set were kept constant, but the proportion of low-SES vs high-SES was varied in the training set.

**FIGURE 9:**
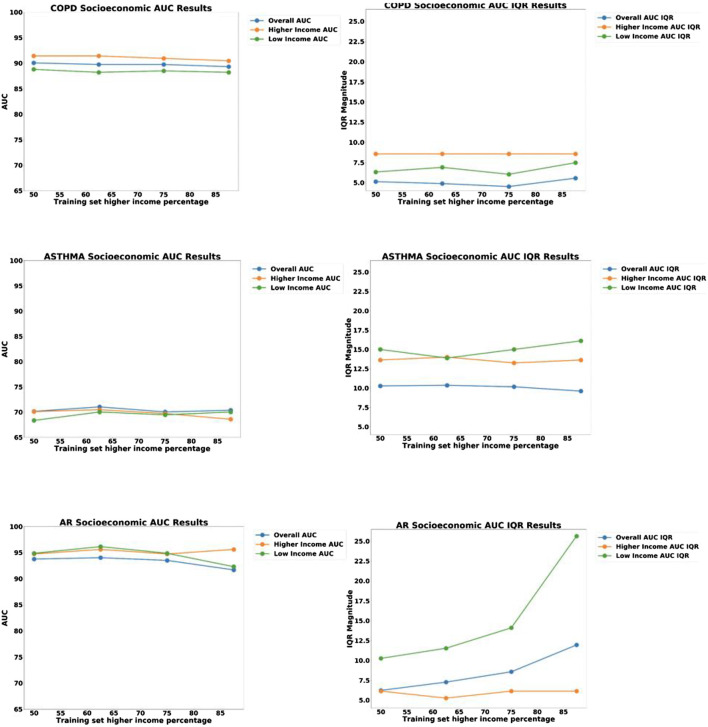
Plots of AUC Accuracy **(left)** and InterQuartile Range **(right)** results of Socio-economic (SES) bias analysis for three different disease diagnostic models: **(top)** COPD; **(middle)** Asthma; and **(bottom)** Allergic Rhinitis (AR). The three colored lines in each plot represent the AUC accuracy for: all groups, High-SES only, and Low-SES only, respectively. The horizontal axis represents the proportion of high-SES patients in the training set, ranging from 50to 100%.

We then proceeded to examine the presence of bias due to sampling by creating a held-out test set of 58 patients consisting of equal proportion of high-SES and low-SES. From the remaining 245 patients, we created four different pools of training data having different proportions of low-SES and high-SES: 50% low-SES, 37.5% low-SES, 25% low-SES, and 12.5% low-SES. For each iteration of the analysis, a different set of patients outside the test set would be randomly selected to be part of the N = 140 training set. One thousand iterations of models were computed and the median and IQR variation was recorded. The data partitioning for each experiment are shown graphically in Figure 8, and the results of 1,000 iterations of this analysis are shown in Figure 9.

Looking at the results in Figure 7, we can see that the ROC curves for the low-SES and high-SES groups are qualitatively similar for all diseases. However, we do see that for COPD, the AUC value is moderately higher for high-SES compared to low-SES.

In the sampling bias analysis (Figure 9), we can see that the accuracy remains fairly consistent for each disease as the proportion of low-SES patients is varied, which indicates that sampling in the training set is not a cause of bias. In the variability plots, however, we observed that the AUC for Allergic Rhinitis (AR) has increased variability as the proportion of low-SES patients is reduced.

#### Discussion and Investigation of Bias Results

Although the machine learning model exhibited no significant bias for the Asthma and AR disease models, a significant diagnostic bias was noted for the COPD in terms of gender and also for socio-economic status (SES). Since this bias persists even when the training data is equally divided among demographic groups, we know that this is not due to sampling bias in the training data. What then is the cause of this bias?

In order to explore this further, we then explored the various risk factors for COPD and examined if any of these factors could be dependent on demographic group. It is well-known that one of the greatest risk factors for COPD is smoking cigarettes (a cause of emphysema that contributes to COPD), which is not true of Asthma. This was also confirmed by performing a coefficient analysis on the logistic regression model and noting that the coefficient value of the smoking variable was relatively large.

**FIGURE 10:**
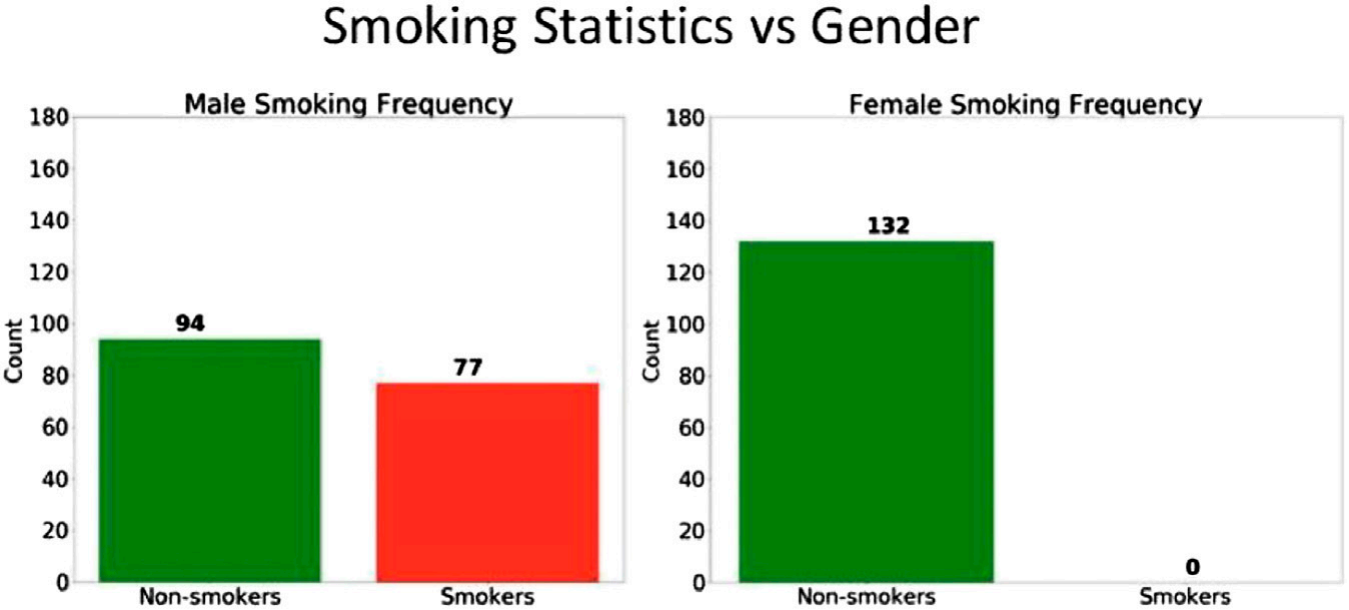
The number of smokers and non-smokers in the each gender group.

Upon further analysis of the patient data, it was discovered that the smoking behavior between men and women was indeed quite different. As shown in Figure 10, approximately 55% of men smoked cigarettes, whereas none of the female subjects used cigarettes. From this observation, we can hypothesize that the gender bias in the algorithms are primarily due to the large disparity in the smoking status between men and women. Other features did not show any significant gender disparity.

The smoking data also helps explain why the model accuracy is higher for women compared to men. Since none of the female patients smoke cigarettes, there is less variance in the features of the female patient population, and thus the model is better able to predict COPD and achieve a higher accuracy.

**FIGURE 11:**
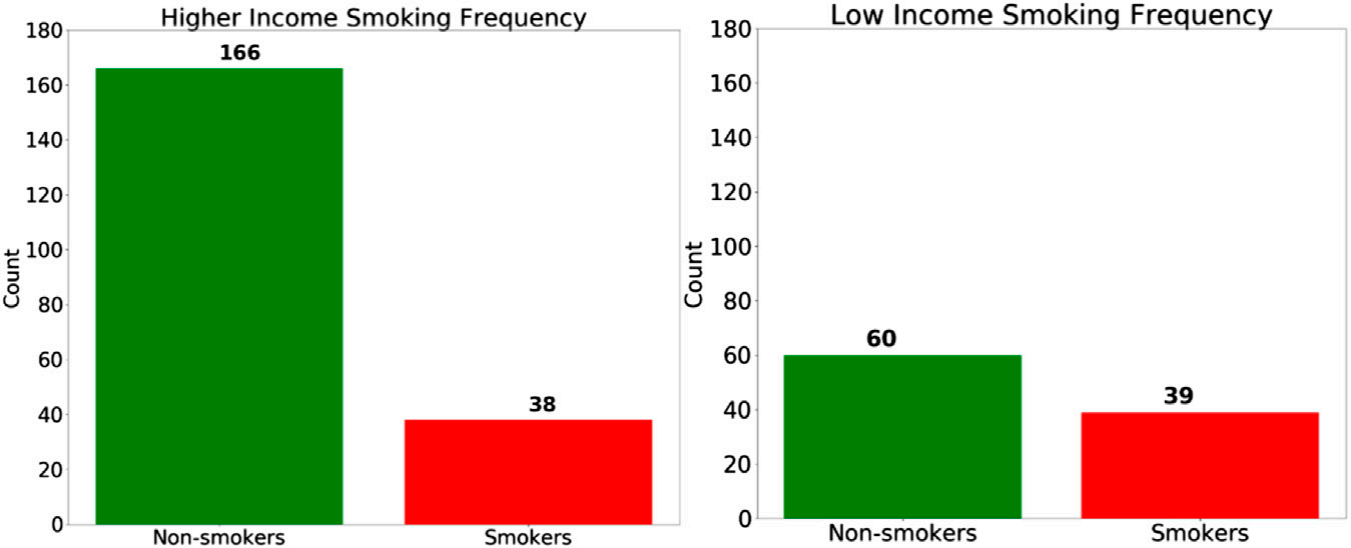
The number of smokers and non-smokers in the each Socio-economic status (SES) group.

Regarding the SES bias analysis in the COPD model, we also investigated how the smoking behavior differs for the two groups (Figure 11), and we discovered that the high-SES group is predominantly comprised of non-smokers, whereas the low-SES group is roughly evenly divided between smokers and non-smokers. Based on this observation, we can also hypothesize that the small disparity in the accuracy between high-SES and low-SES patients is primarily due to the disparity in the smoking prevalence among these patients’ groups. As with the case of the female patients in the gender bias analysis, we can also see that the COPD model produces a higher accuracy among high-SES patients, mostly likely because this patient group is more homogeneous in terms of smoking status.

This bias analysis suggests that it could be better to stratify the patients, and deploy a separate COPD model for women vs. men, or perhaps a separate model for smokers vs. non-smokers. However, in our case, we were not allowed to deploy a different model for women vs. men, so a single model had to be used. While a separate model for smokers was not useful in our case, there are other applications, such as predicting health insurance premiums, where smokers could be treated as a separate group. Rather than charging a higher insurance premium to all males, it would be possible to specifically identify the smoking behavior and only penalize patients who have that behavior.

Another result worth noting is the stability of the AR disease model as a function of SES. We notice in Figure 9 that the IQR variability of the model increases significantly if the proportion of low-SES patients is reduced. This demonstrates that the AR disease model benefits from having more diverse representation from all SES groups.

### Fairness Considerations

#### Understanding Bias to Improve Fairness

As shown in Figure 2, our COPD prediction model produced qualitatively different ROC curves for males vs. females, and a similar but less significant bias was found for COPD with respect to the patient SES. Without addressing these biases, our COPD prediction model could produce problems of unfairness.

As discussed above, the cause of bias in this case is due to the inherent properties of the patients themselves (i.e., smoking behavior) and therefore, cannot be mitigated. In order to enforce fairness in the presence of bias, we needed to choose a definition of fairness that we could enforce in the algorithm. As discussed in *Definitions of Fairness* section, we chose the *equality of odds* fairness criteria, and we tuned the threshold of the model to the point where the ROC curve for males intersects with the ROC curve for females. This change created a slight reduction in the classification accuracy for females but produced equal results for both genders, with a classification accuracy of approximately 89.2%. This change also produced similar results for the two SES demographic groups in the COPD model.

### Final Tuning for Model Deployment

The final step in our machine learning model development was to adjusting the baseline probability in each of our models to match the prevalence in the target population. For implementing our model in the clinical setting, it was necessary to know the prevalence of each of the disease categories that is observed in the clinic. Since this data is not generally published anywhere, we recorded the diagnosis of 200 consecutive patients at the main clinic, and we used this data to estimate the prevalence for each model. Adjustment to the logistic regression zero-order coefficient was made as discussed in *Bias* section above.

The final version of the model was then incorporated into an Android mobile application for use by the local GP doctors. A description of a similar mobile application for general use has been published previously (Anand et al., 2018).

## Conclusion and Recommendations

### Machine Learning Challenges in Global Health

The introduction and case study presented in this paper are focused on the issues of Fairness, Bias and Appropriateness. These issues are not unique to LMICs, but are particularly important in developing countries, where there may not exist a legal framework to regulate machine learning or enforcement to prevent discrimination between different demographic groups. The astounding economic disparities that exist in many developing countries also present severe challenges in maintaining fair access and benefit from these technologies.

Technology infrastructure, such as electronic medical records (EMR), can facilitate the adoption of machine learning and AI, but it also introduces risks. A recent study in the United States found that machine learning algorithms that rely on electronic health care data tend to discriminate against poor and minority populations that cannot afford frequent and continuous medical care (Obermeyer et al., 2019). Mobile phones now present an interesting opportunity to bring artificial intelligence to new segments of the world’s population, through the use of consumer-facing mobile apps and tools for community health care workers; however, these new platforms will also introduce new risks for misuse and health disparities.

It is also important to note that in addition to issues of Fairness, Bias and Appropriateness, global health certainly faces many additional challenges that need to be considered in the process of adopting AI and machine learning. While the need is very great, other issues of technical capacity, education, public perception, and cultural sensitivity need to be addressed simultaneously as this technology evolves (Paul and Schaefer, 2020).

### Recommendations for Fairness, Bias, and Appropriate Use

In summary, it is evident that the processes we encounter in global health (e.g., disease, and human behavior) are complex and can often include hidden variables. Machine learning analysis generally requires creating crude approximations to these processes, which must be done carefully and with the proper domain expertise, in order to avoid introducing errors and false conclusions. Keeping in mind the preceding discussion, we can formulate a set of basic recommendations that can be used as guidelines for applying artificial intelligence and machine learning in the context of global health:

1. *Question appropriate use:* Machine learning models are designed to answer specific questions. However, since many health care decisions can have unintended consequences, it is always important to review if we are posing the right question. As the use of machine learning and AI is extended beyond diagnostic tools into questions related to health care access, medical triage, and insurance coverage, it is also important to remember that no decision-making tool is perfect, and that certain important decisions should perhaps be reserved for humans and not machines.
2. *Maintain transparency for critical decisions:* As the complexity of machine learning models continues to increase, and new models are invented (e.g., deep neural nets), the ability to explain the decision of a computer may become increasingly challenging. The use of “black box” models is useful in certain cases but not others. For decisions that involve human input, such as patient diagnosis, it is recommended to use interpretable models that will enable review and consensus from human staff.
3. *Enforce transparency in data and algorithms:* Algorithms are not universal, and are only valid when used properly. Algorithms are critically dependent on the specific training data that was used for development, as well as the optimization criteria used to tune the model. In order to avoid problems with fairness, it is important that this information be disclosed to organizations and patients that will be using the model. The government regulation of algorithms is in a very early stage, but it is perhaps inevitable that some type of regulation will be put in place, in a manner analogous to the FDA regulation of pharmaceuticals; “off-label” uses of an algorithm can produce unexpected or unfair results and should be avoided.
4. *Address and Respect Bias:* Bias should be examined at each level of the computation, and the individual features used in the training data should themselves be examined for bias. While the bias found in other application domains of machine learning (finance, employment, law enforcement, etc.) may often be due to sampling bias or implicit cultural bias, the domain of health also contains true systematic bias inherent in biological processes which may not be possible to mitigate or “repair.” In the domain of health, there are true genetic differences across different races and ethnic groups which affect disease prevalence, and these differences cannot (and should not) be ignored. If it is revealed that a particular algorithm consistently produces very different results for one patient group vs. another, it is generally best to design a separate algorithm for each group rather than try to create a universal algorithm that will likely perform poorly on both groups.
5. *Agree on a Fairness Metric:* Since Fairness can be defined in multiple ways, it is important in each case, to decide which fairness metric will be applied and agree on the criteria that are being optimized. It is important to recognize that this step involves much more than technical expertise, and requires the participation of all stakeholders, including the individual groups that may be impacted by the use of the algorithm. While individual fairness is a good ideal, most laws are written with respect to group fairness, and thus when machine learning decisions are applied across multiple groups, it should be recognized that trade-offs and compromises will often need to be made, to reconcile how the benefit and the risk will be shared across all groups.

In this paper, we have presented some important considerations and guidelines that should be examined whenever machine learning is being applied to health applications in all phases of the project lifecycle. The use of artificial intelligence and machine learning to health can bring enormous benefits, but this domain is complex, and such algorithms should be designed with the proper considerations and domain expertise required to ensure that the ultimate goals of applying the algorithms are safely met without creating any harm to all parties involved.

## Data Availability

The raw data supporting the conclusions of this article will be made available by the authors, without undue reservation.
